# Supplementary material for: A comparison of several media types and basic techniques used to assess outdoor airborne fungi in Melbourne, Australia
Source: PLoS One. 2020 Dec 18;15(12):e0238901. doi: 10.1371/journal.pone.0238901 (PMC7748268; doi:10.1371/journal.pone.0238901)
Supplement: S1 File — These tables include CFU/plate counted, calculated CFU/m3 air via Andersen, 1958 with a 1.25x adjustment to the raw CFU/plate given polymer Petri dishes were used, and general categories of genera as adapted from ASTM D7391-20 section 12.3.2 [78], pertaining to Figs 1–9 and Table 1. (DOCX) [file pone.0238901.s001.docx]

***S1 File: Tables of raw data.***

These tables include CFU/plate counted, calculated CFU/m3 air via Andersen, 1958 with a 1.25x adjustment to the raw CFU/plate given polymer Petri dishes were used, and general categories of genera as adapted from ASTM D7391-20 section 12.3.2 [73].

**Table S1.1 (Fig 1): Media Tests, maltose**

Media: 1. SabCG from scratch (SBCG 2120, 4433); 2. PeptoneCG (MPCG); 3. AgarCG (ACG); 4. MaltoseCG (MCGA); 5. PeptoneMaltoseCG (SMCG); 6. PeptoneMaltoseGlucoseCG (SABM).

**Table S1.1 (Fig 1):** Media Tests, maltoseCG, SabCG (= glucose-peptoneCG), PeptoneCG, agarCG, maltose-glucoseCG,

| **Media ID** | **Colony Forming Units**  **(CFU)** | | **Identified fungi** (#CFU) | | | | | | | | | | | | | | | | | | | |
| --- | --- | --- | --- | --- | --- | --- | --- | --- | --- | --- | --- | --- | --- | --- | --- | --- | --- | --- | --- | --- | --- | --- |
|  |  |  | 1. Alternaria \ Ulocladium  11. Ulocladium | 2. ascomycete (undiff.) | 3. Aspergillus | 3. Penicillium | 4. basidiomycete  (undiff.) | | 6. Cladosporium | | 7. Curvularia | | X. Trichoderma | | X. Chrysonilia | | X.Plant. phyllo/Phoma | | | X: yeasts, | | X. Zygomycetes  Rhyz/Muc. |
| **AIRBORNE AV** | **/samp-le** | **/m^3^** |  | | | | | | | | | | | | | | | | | | | |
| 1.1 SBCG from stock | 119 | 993 | 30 | 50 | 2 | 17 |  | 14 | |  | |  | |  | |  | | | 6 | |  | |
| 1.2 SBCG stock | 103 | 860 | 6 | 63 | 1 | 13 |  | 19 | |  | |  | |  | |  | | |  | | 1 | |
| 1.3 SBCG stock | 146 | 1293 | 30 | 65 | 1 | 29 |  | 18 | |  | |  | |  | | 1 | | | 2 | |  | |
| 1 mean, SD | 123,22 | 1049, 222 |  |  |  |  |  |  | |  | |  | |  | |  | | |  | |  | |
| 2.1 SabCG from scratch | 168 | 1987 | 33 |  | 1 | 25 |  | 33 | |  | |  | |  | | 1 | | | 7 | |  | |
| 2.2 SabCG scratch | 83 | 720 | 19 |  |  | 15 |  | 16 | |  | |  | |  | |  | | | 8 | | 1 | |
| 2.3 SabCG scratch | 54 | 500 | 10 |  | 2 | 7 |  | 4 | |  | |  | |  | |  | | | 4 | |  | |
| 2 mean, SD | 102, 59 | 1069, 803 |  |  |  |  |  |  | |  | |  | |  | |  | | |  | |  | |
| 3.1 MPCG peptone-CG | 120 | 1000 | 11 |  |  | 14 |  | 35 | |  | |  | |  | |  | | | 15 | |  | |
| 3.2 MPCG | 54 | 500 | 15 |  |  | 10 |  | 9 | |  | |  | |  | |  | | | 5 | | 1 | |
| 3.3 MPCG | 64 | 593 | 9 |  | 1 | 9 |  | 10 | |  | |  | |  | |  | | | 2 | |  | |
| 3 mean, SD | 79,36 | 698, 266 |  |  |  |  |  |  | |  | |  | |  | |  | | |  | |  | |
| 4.1 ACG agar-cg *1 | (6) | (53) |  | (6) |  |  |  |  | |  | |  | |  | |  | | |  | |  | |
| 4.2 ACG | (8) | (73) |  | (8) |  |  |  |  | |  | |  | |  | |  | | |  | |  | |
| 4.3 ACG | (7) | (67) |  | (7) |  |  |  |  | |  | |  | |  | |  | | |  | |  | |
| 4 mean, SD | (0, 0) | (0, 0) |  |  |  |  |  |  | |  | |  | |  | |  | | |  | |  | |
| 5 MCGA maltose-cg *1 | (5) | (40) | (2) | (3) |  |  |  |  | |  | |  | |  | |  | | |  | |  | |
|  | (9) | (80) | (5) | (3) |  |  |  |  | |  | |  | |  | |  | | (1) | | |  | |
|  | (8) | (73) | (4) | (3) |  |  |  |  | |  | |  | |  | |  | | (2) | | |  | |
|  | (0, 0) | (0, 0) |  |  |  |  |  |  | |  | |  | |  | |  | |  | | |  | |
| 6 SMCG maltose-peptone.CG | 64 | 593 | 15 |  | 2 | 18 |  | 19 | |  | |  | | 1 | | 1 | | 13 | | |  | |
|  | 66 | 620 | 13 |  | 2 | 10 |  | 23 | |  | |  | |  | |  | | 7 | | |  | |
|  | 59 | 547 | 12 |  | 1 | 14 |  | 13 | |  | |  | | OG | |  | | ? | | |  | |
|  | 63, 4 | 587, 37 |  |  |  |  |  |  | |  | |  | |  | |  | |  | | |  | |
| 7.1 SABM peptone-glucose-maltose.CG | 83 | 693 | 15 |  |  | 17 |  | 25 | |  | |  | | 1 | |  | | 6 | | |  | |
| 7.2 SABM | 64 | 593 | 5 |  | 2 | 13 |  | 6 | |  | |  | |  | |  | | ? | | | 4 OG | |
| 7.3 SABM | 71 | 673 | 2 |  |  | 19 |  | 29 | |  | |  | | 1 OG | |  | | 7 | | |  | |
| 7 mean, SD | 73, 10 | 653, 53 |  |  |  |  |  |  | |  | |  | |  | |  | |  | | |  | |

**Table S1.2 (Figs 2 and 3):** Clarified V8, Tomato and celery juices with/without heat treatment (17-20 April 2020)

| Medium | CFU/plate | /m^3 |
| --- | --- | --- |
| control TJc uninoculated ui | 0 | 0 |
| control TJr ui | 2 | 20 |
| control V8r ui | 0 | 0 |
| control V8c ui | 0 | 0 |
| control CJc ui | 3 | 27 |
| control CJr ui | 0 | 0 |
| SabCG 1 | 34 | 300 |
| SabCG 2 | 30 | 267 |
| SabCG 3 | 34 | 300 |
| TJr 3 | 43 | 387 |
| TJr 2 | 28 | 247 |
| TJr 1 | 34 | 300 |
| TJc 1 | 33 | 287 |
| TJc 2 | 39 | 347 |
| Tjc 3 | OG | N/A |
| V8r 3 | OG | N/A |
| V8r 2 | 28 | 247 |
| V8r 1 | 35 | 307 |
| V8c 1 | 22 | 200 |
| V8c 2 | 36 | 313 |
| V8c 3 | 40 | 353 |
| CJr 1 | 38 | 340 |
| CJr 2 | 38 | 340 |
| CJr 3 | 34 | 300 |
| CJc 1 | ~ N/A | N/A |
| CJc 2 | 30 | 267 |
| CJc 3 | 45 | 400 |

**Table S1.4 (Figs 6 and 7):** Mineral supplemented media MS1 and MS2, Sabouraud, MEA, PDA with/without CG antibiotics, seeded with bacteria powder.

| **Media ID** | **Colony Forming Units**  **(CFU)** | | **Identified fungi** (by coverage; adapted from ASTM D7658-17, D7391-17) | | | | | | | | | | | | | | | | | | | |
| --- | --- | --- | --- | --- | --- | --- | --- | --- | --- | --- | --- | --- | --- | --- | --- | --- | --- | --- | --- | --- | --- | --- |
|  |  |  | 1. Alternaria \  11. Ulocladium | 2. ascomycete (undiff.) | 3. Aspergillus | 3. Penicillium | 4. basidiomycete  (undiff.) | | 6. Cladosporium | | 7. Curvularia | | X. Trichoderma | | X. Chrysonilia | | X.Plant. phyllo/Phoma | | | X: yeasts, bacteria | | X. Zygomycetes  Rhyz/Muc. |
| **AIRBORNE AV** | **/samp-le** | **/m^3^** |  | | | | | | | | | | | | | | | | | | | |
| 1.1 Mineral Supp BLKS3396 halos | 6 (15) | 125  2min*1 |  | + |  | + |  | ++* | |  | |  | |  | |  | | |  | |  | |
| 1.2 halos | 30 | 267 |  | ++ | ? | ++ |  | ++* | |  | |  | |  | |  | | |  | |  | |
| 1.3 halos *2 | (350) | (17520) | ++ | ++ |  | ++++ *** |  | +++* | |  | |  | |  | | + | | |  | |  | |
| 1.4 halos | 31 | 273 |  | ++ |  | ++ |  | ++ | |  | |  | |  | |  | | |  | |  | |
| 1. Mean, SD | 25, 9 | 222, 84 |  |  |  |  |  |  | |  | |  | |  | |  | | |  | |  | |
| 2.1 Mineral Supp BLK3397 | 36 | 313 | +++ | ++ | + nig | +++ |  | ++ | |  | |  | |  | |  | | | + | |  | |
| 2.2 halos | 39 | 347 | +++ | ++ | ++ | +++ |  | ++ | |  | |  | |  | |  | | |  | |  | |
| 2.3 | 34 | 300 | +++ | ++ |  | ++ |  | + | |  | |  | |  | |  | | | + | |  | |
| 2 Mean, SD | 36, 3 | 320, 24 |  |  |  |  |  |  | |  | |  | |  | |  | | |  | |  | |
| 3.1 Sabouraud +CG SBCG3075 | 35 | 307 | +++ | ++ |  | ++ |  | +++ | |  | |  | |  | |  | | |  | |  | |
| 3.2 halos | 30 | 267 | +++ | ++* |  | ++ |  | ++ | |  | |  | |  | |  | | |  | |  | |
| 3.3 halos | 31 | 273 | ++ | ++ |  | ++ |  | ++ | |  | |  | |  | |  | | |  | |  | |
| 3. Mean, SD | 32, 3 | 282, 22 |  |  |  |  |  |  | |  | |  | |  | |  | | |  | |  | |
| 4.1 MEA +CG MECG3349 | 35 | 307 | + | ++ |  | ++ |  | + | |  | |  | |  | |  | | + | | |  | |
| 4.2 | 23 | 207 | ++ | ++ |  | + |  | ++ | |  | |  | |  | |  | |  | | |  | |
| 4.3 | 19 | 167 | ++ | + |  | ++ |  | + | |  | |  | |  | |  | | + | | |  | |
| 4 Mean, SD | 26, 8 | 227, 72 |  |  |  |  |  |  | |  | |  | |  | |  | |  | | |  | |
| 5.1 MEA -  MEA3348 | 31 | 273 | + | ++ |  | ++ |  | + | |  | |  | |  | |  | | ? + X1 | | |  | |
| 5.2 | 29 | 253 | ++ | ++ |  | ++ |  | + | |  | |  | |  | |  | | ? + X1 | | |  | |
| 5.3 | 17 | 147 | + | + |  | + |  | + | |  | |  | |  | |  | | + blk wht  X1 | | | 5 OG | |
| 5 Mean, SD | 26, 8 | 233, 61 |  |  |  |  |  |  | |  | |  | |  | |  | |  | | |  | |
| 6.1 PDA +CG  PDCG3330 | 34 | 300 | ++ | + |  | ++* |  | + | |  | |  | |  | |  | |  | | |  | |
| 6.2 | 20 | 180 | ++ | + |  | + |  | ++ | |  | |  | |  | |  | | + | | |  | |
| 6.3 | 25 | 220 | ++ | ++ |  | ++ |  | + | |  | |  | |  | |  | | + | | |  | |
| 6 Mean, SD | 27, 7 | 233, 61 |  |  |  |  |  |  | |  | |  | |  | |  | |  | | |  | |
| 7.1 PDA -  PDA3178 | 32 | 280 | + | ++ | + | +++ |  |  | |  | |  | |  | |  | | + X1 | | |  | |
| 7.2 | 13 | 113 | ++ | ++ |  | + |  | + | |  | |  | |  | |  | | X1 | | |  | |
| 7.3 | 20 | 180 | ++ | ++ | + nig | + |  | + | |  | |  | | 1 | |  | | +? X1 | | |  | |
| 7 Mean, SD | 22, 10 | 191, 84 |  |  |  |  |  |  | |  | |  | |  | |  | |  | | |  | |

5sept19 and 11sept19: All were seeded with bacteria powder (airborne)

X1: many tiny buff slimy/shiny colonies in 400-hole pattern. Bacteria. Appear dark against bright background

*1: run ended prematurely due to battery problems. All other runs used mains charger + battery. Converted to likely #CFU if allowed to run 5 mins.

*2: transient spike in Penicillium, possibly accidental contamination. Excluded from average, SD

‘halo’: noted that zones of clearing had occurred around colonies. The media was originally somewhat cloudy, presumably a mineral precipitate that dissolved with carbon dioxide or other organic compounds from the colony. Media left in ‘fridge for many weeks cleared by itself. Possibly carbon dioxide in that case.

**Table S1.5 (Figs 8 and 9):** Various glucose concentrations, 0-8% with PeptoneCG, SabCG (from stock, = 4% glucose) and GlucoseCG controls.

| **Media ID** | **Colony Forming Units**  **(CFU)** | | **Identified fungi** (#CFU) | | | | | | | | | | | | | | | | | | |
| --- | --- | --- | --- | --- | --- | --- | --- | --- | --- | --- | --- | --- | --- | --- | --- | --- | --- | --- | --- | --- | --- |
|  |  |  | 1. Alternaria \  11. Ulocladium | 2. ascomycete (undiff.) | 3. Aspergillus | 3. Penicillium | 4. basidiomycete  (undiff.) | | 6. Cladosporium | | 7. Curvularia | | X. Trichoderma | | X. Chrysonilia | | X.Plant. phyllo/Phoma | | X: yeasts, | | X.Zygomycetes  Rhyz/Muc. |
| **AIRBORNE AV** | **/samp-le** | **/m^3^** |  | | | | | | | | | | | | | | | | | | |
| 1-1 SabCG stock 347 x030420 | 36 | 313 | 9 | 2 | 2 fum | 6 |  | 15 | |  | |  | |  | | 1 | | 1 | |  | |
| 1-2 | 33 | 287 | 8 | 9 | 2 | 2 |  | 10 | |  | |  | |  | |  | | 2 | |  | |
| 1-3 | 39 | 347 | 7 | 6 | 1 | 9 |  | 11 | |  | |  | |  | | 2 | | 3 | |  | |
| 1-mean, SD | 36, 3 | 316, 30 |  |  |  |  |  |  | |  | |  | |  | |  | |  | |  | |
| 2-1 Glucose-only 469 x070420 | (16) | (140) |  | (16) |  |  |  |  | |  | |  | |  | |  | |  | |  | |
| 2-2 | (9) | (80) |  | (9) |  |  |  |  | |  | |  | |  | |  | |  | |  | |
| 2-3 | (4) v small, undevelop | (33) |  | (4) |  |  |  |  | |  | |  | |  | |  | |  | |  | |
| 2-mean, SD | 10, 6 | 84, 54 |  |  |  |  |  |  | |  | |  | |  | |  | |  | |  | |
| 3-1 no gluc, peptone only 470 x070420 | 23 small | 207 |  | 16 |  | 7 moderate size + small |  |  | |  | |  | |  | |  | |  | |  | |
| 3-2 | 12 | 107 |  | 7 |  | 5 mod |  |  | |  | |  | |  | |  | |  | |  | |
| 3-3 | 28 | 247 | 4 | 5 | 7 nig fum ter | 5 small |  | 6 v small | |  | |  | |  | |  | | 1 v small | |  | |
| 3-mean, SD | 21, 8 | 187, 72 |  |  |  |  |  |  | |  | |  | |  | |  | |  | |  | |
| 4-1 1%gluc 471 SAB1 | 25 | 220 |  | 9 | 2 fum | 14 |  |  | |  | |  | |  | |  | |  | |  | |
| 4-2 | 27 | 240 | 1 | 15 | 1 fum | 7 |  |  | |  | |  | | 1 | |  | | 2 | |  | |
| 4-3 | 33 | 287 | 1 | 21 |  | 5 |  | 1 | |  | |  | | 1 | |  | | 4 | |  | |
| 4-mean, SD | 28, 4 | 249, 34 |  |  |  |  |  |  | |  | |  | |  | |  | |  | |  | |
| 5-1 2%gluc 472 SAB2 | 35 | 307 |  | 20 | 1 ter | 7 |  | 5 | |  | |  | |  | |  | | 2 | |  | |
| 5-2 | 26 | 233 | 2 | 12 | 2 fum | 4 |  | 1 | |  | |  | |  | | 2 | | 3 | |  | |
| 5-3 | 38 | 340 |  | 23 | 2 fum nig | 6 |  | 2 | |  | |  | | 2 | |  | | 3 | |  | |
| 5-mean, SD | 33, 6 | 293, 55 |  |  |  |  |  |  | |  | |  | |  | |  | |  | |  | |
| 6-1 4%gluc 473 SAB4 | 22 | 200 | 2 | 5 | 2 fum | 6 |  |  | |  | |  | |  | |  | | 7 | |  | |
| 6-2 | 26 | 233 | 1 | 6 | 3 | 9 |  | 2 | |  | |  | |  | |  | | 5 | |  | |
| 6-3 | 23 | 207 | 2 | 12 | 1 | 5 |  |  | |  | |  | |  | |  | | 3 | |  | |
| 6-mean, SD | 24.2 | 213, 17 |  |  |  |  |  |  | |  | |  | |  | |  | |  | |  | |
| 7-1 8%gluc SAB8 | 27 | 240 |  | 16 | 4 nig fum ter | 2 |  | 1 | |  | |  | |  | |  | | 4 | |  | |
| 7-2 | 35 | 307 | 2 | 18 | 1 ter | 5 |  | 1 | |  | |  | |  | |  | | 8 | |  | |
| 7-3 | 36 | 313 | 1 | 21 | 4 nig fum | 5 |  | 1 | |  | |  | |  | |  | | 4 | |  | |
| 7-mean, SD | 33,5 | 287, 41 |  |  |  |  |  |  | |  | |  | |  | |  | |  | |  | |

Table S1.3 (Figs 4 and 5): DG18C, SabCG, V8C, six-replicate plates, 150 L outdoor air (30 July 2020, winter)

| **Media ID** | **Colony Forming Units**  **(CFU)** | | **Identified fungi** (#CFU) | | | | | | | | | | | | | | | | | | |
| --- | --- | --- | --- | --- | --- | --- | --- | --- | --- | --- | --- | --- | --- | --- | --- | --- | --- | --- | --- | --- | --- |
|  |  |  | 1. Alternaria \ 11. Ulocladium | 2. ascomycete (undiff.) | 3. Aspergillus | 3. Penicillium | 4. basidiomycete  (undiff.) | | 6. Cladosporium | | 7. Curvularia | | X. Trichoderma | | X. Chrysonilia | | X.Plant. phyllo/Phoma | | X: yeasts, | | X. Zygomycetes  Rhyz/Muc. |
| **AIRBORNE AV** | **/samp-le** | **/m^3^** |  | | | | | | | | | | | | | | | | | | |
| 1.1 SBCG (Sabouraud-CG) | 21 | 187 |  | 7 |  | 10 |  | 4 | |  | |  | |  | |  | |  | |  | |
| 1.2 | 23 | 207 | 3 | 5 |  | 5 |  | 10 | |  | |  | |  | |  | |  | |  | |
| 1.3 | 41 | 367 |  | 8 | 1 nig | 2 |  | 28 | |  | |  | |  | |  | | 2 w | |  | |
| 1.4 | 21 | 187 | 1 | 5 |  | 10 |  | 5 | |  | |  | |  | |  | |  | |  | |
| 1.5 | 26 | 233 | 1 | 14 | 1 ter | 7 |  | 4 | |  | |  | |  | |  | |  | |  | |
| 1.6 | 31 | 273 |  | 10 | 1 ter  1 - | 8 |  | 8 | |  | |  | |  | |  | | 3 w | |  | |
| 2.1 DG18C (DG18 with Chloramphenicol/Gentamycin CG) | 25 | 220 |  | 5 |  | 12 |  | 8 | |  | |  | |  | |  | |  | |  | |
| 2.2 | 12 | 107 |  | 5 |  | 5 |  | 2 | |  | |  | |  | |  | |  | |  | |
| 2.3 | 15 | 133 |  | 7 |  | 4 |  | 4 | |  | |  | |  | |  | |  | |  | |
| 2.4 | 12 | 107 | 1 |  | 1 * | 4 |  | 6 | |  | |  | |  | |  | |  | |  | |
| 2.5 | ~31 ^ | 273 |  | 7 |  | 7 |  | 4 | |  | |  | |  | |  | | ~13 | |  | |
| 2.6 | 21 | 187 | 2 * | 6 |  | 6 |  | 7 | |  | |  | |  | |  | |  | |  | |
| 3.1 V8CG (full-strength whole V8 medium with CG) | 7 (4a) | 67 | 2a | 3a |  | 2 * |  |  | |  | |  | |  | |  | |  | |  | |
| 3.2 | 10 (6a) | 93 | 4 | 1-  3a | 1a | 1a |  |  | |  | |  | |  | |  | |  | |  | |
| 3.3 | 4 (2-) (2a) | 33 |  | 2a |  |  |  | 2-* | |  | |  | |  | |  | |  | |  | |
| 3.4 | 9 (5a) | 80 | 3 |  | 3a | 2* |  | 1a | |  | |  | |  | |  | |  | |  | |
| 3.5 | 6 (1a) | 53 |  | 1a  1-* |  | 1-* |  | 3- | |  | |  | |  | |  | |  | |  | |
| 3.6 | 13 (2a) | 113 | 1a | 1-* |  | 1a  3-* |  | 7-* | |  | |  | |  | |  | |  | |  | |

* really small colonies

Notes: colonies on DG18c smaller, less developed than SabCG. More difficult to ID

“a”: zone of clearing in V8 (often even without staining), and doesn’t stain with Lugol’s Iodine (I/KI), c.f., surrounding gel, hence amylase positive. Noted that colonies are virtually always either large/a+, OR small/a-, presumably because they are sugar-starved? Iodine-starch dark colour fades after a few hours.

^: suspiciously high. Suspected yeast contamination in condensate droplet that ran over the gel surface.
