# Supplementary material for: A comparison of several media types and basic techniques used to assess outdoor airborne fungi in Melbourne, Australia
Source: PLoS One. 2020 Dec 18;15(12):e0238901. doi: 10.1371/journal.pone.0238901 (PMC7748268; doi:10.1371/journal.pone.0238901)
Supplement: Attachment — Submitted filename: Response to Reviewers 2 - 17nov2020.docx [file pone.0238901.s004.docx]

Response to Reviewers

WD Black (WDB), 17-24 Nov 2020

Noted: all other sections both reviewers affirm compliance/acceptance, except Section 6 in which this is either unclear or noted as a helpful suggestion.

I thank the reviewers for their comments and attempt to address them below.

***

6. Review Comments to the Author

Please use the space provided to explain your answers to the questions above. You may also include additional comments for the author, including concerns about dual publication, research ethics, or publication ethics. (Please upload your review as an attachment if it exceeds 20,000 characters)

***Reviewer #1:*** *Overall the author did a good job addressing my comments.

There is still only one issue that need to be addressed:

Introduction: I think the author can make the introduction even shorter; I see your need in outlying the framework and prior research; but I think that in order to attract the reader to read the paper, you want to get to your main point earlier; I do not have specific suggestions how to do it; and you don’t have to agree with this suggestion, of course; it is just a suggestion to enhance readability of the paper.*

I thank Reviewer #1 for their suggestions.

I have attempted to address these by a significant ‘haircut’ of the introduction to weed out material I realise was written with a non-scientific audience in mind. I realise they’d be unlikely to read the paper in any guise, and can better make my point to them by other means such as PowerPoint and merely referring to the paper.

The main concern I have is that I will be criticised for not thoroughly reviewing the literature, and also not framing the problems I attempt to address in the series of experiments I performed. If you see this is OK as-is now, then all good.

There are of course a number of questions asked of me by that non-scientific audience I was hoping to answer in this more formal scientific forum by way of the longer introduction. I think I have achieved a balance here in cutting it down to 1800 words or so, trimming out 80 lines.

I’ve repositioned some sentences to better streamline the reading flow.

***Reviewer #2:*** *I thank the author again, the author answered most of my concerns about the MS, traying his best to make the MS more clear and acceptable, I was concern about the conditions of the experiments conducted like small number of replicates and samples size which cause high variation in results within the same media. I think he has made an expressed work in his MS. All the materials and methods are clear now, deleting some photos is possible but at the same time providing good photos for the key experiments will be much appreciated for publication, any possible improvement with more investigations as you indicated in your answers will be also highly appreciated and add much value to this paper.*

I again thank Reviewer #2 for their valuable comments.

To explain, I found it difficult to compare many different media on the same day because of how quickly and dramatically Melbourne weather changes. It is famous for this. So I was obliged to limit the total number of plates per day to best have everything as similar as possible, highlighting any differences in media rather than weather.

I have tried to not burden the readers with too much statistical data, instead using graphs that hopefully tell the story in a readily accessible way, especially the non-scientific readers I am expecting.

In the revised latest manuscript (attached) some p-values are on lines 283-285 (p = 0.0089, three replicate plates), and line 367 (p < 0.0003, six replicate plates), suggesting that the null-hypothesis (H0) of there being no real difference between these media tested, being merely random chance is less than 1 in 113 and 1 in 3333, respectively. Obviously this is better than the often cited p < 0.05 limit, being 1 in 20.

I am therefore fairly confident that these experiments have demonstrated a significant difference between different media as described/presented even at three replicate plate numbers.

I hope I have shown that some of the media are clearly not suitable for this purpose even with three replicate plates only, and logically supported by the evidence that not all random wild airborne fungi are able to digest starch and/or maltose, and hence the use of PDA, V8, MEA is not supported.

I have added the calculated P-value for Fig 2 (lines 322-323), and a comment on line 412.

A quick check of my data indicates that when one takes the six SabCG replicates in groups of three in every combination, the SDs/means vary around the mean by 36%, whereas the full set of six varies by 29%, a difference of 7%. As the number of replicate samples increases, the difference becomes less, but asymptotically, approaching zero but never reaching it.

I suppose when there is a large difference in the media via the number of CFU, one needs fewer replicate plates to see this difference. This is also visible in the figures wherein the +/-SD bars often do not overlap.

Some data is not really able to be meaningfully compared by these means, being more qualitative such as the media clouding (the two mineral supplemented media) or colonies being strangely shaped and under-developed when bacteria are present in number (antibiotic-free media)..

As mentioned, I hope to move beyond investigating the media, and into investigating the differences between mouldy and non-mouldy houses, and the accuracy/precision of test methods using the best candidate medium, SabCG. I will be using more replicates where possible, of course.

I noticed that the quality of the images embedded in the .pdf sent to reviewers are not at the high quality of the original images. I suspect that the final version will be of high enough quality for the readers to see beyond the light reflections, and I hope they also understand these images are really just ‘examples’ rather than the actual data. A little embarrassing for me I admit. I was more focused on the colonies rather than the image itself.
